# Interactions of *Galleria mellonella* Proline-Rich Antimicrobial Peptides with Gram-Negative and Gram-Positive Bacteria

**DOI:** 10.3390/ijms26178438

**Published:** 2025-08-29

**Authors:** Agnieszka Zdybicka-Barabas, Sylwia Stączek, Paweł Mak, Justyna Kapral-Piotrowska, Krzysztof Skrzypiec, Jerzy Wydrych, Bożena Pawlikowska-Pawlęga, Wiesław I. Gruszecki, Małgorzata Cytryńska

**Affiliations:** 1 Department of Immunobiology, Institute of Biological Sciences, Faculty of Biology and Biotechnology, Maria Curie-Skłodowska University, Akademicka 19 St., 20-033 Lublin, Poland; sylwia.staczek@mail.umcs.pl (S.S.); malgorzata.cytrynska@mail.umcs.pl (M.C.); 2 Department of Analytical Biochemistry, Faculty of Biochemistry, Biophysics, and Biotechnology, Jagiellonian University, Gronostajowa 7 St., 30-387 Krakow, Poland; pawel.mak@uj.edu.pl; 3 Department of Functional Anatomy and Cytobiology, Institute of Biological Sciences, Faculty of Biology and Biotechnology, Maria Curie-Skłodowska University, Akademicka 19 St., 20-033 Lublin, Poland; justyna.kapral-piotrowska@mail.umcs.pl (J.K.-P.); jerzy.wydrych@mail.umcs.pl (J.W.); bozena.pawlikowska-pawlega@mail.umcs.pl (B.P.-P.); 4 Analytical Laboratory, Faculty of Chemistry, Maria Curie-Skłodowska University, M.C. Skłodowska Square 5, 20-031 Lublin, Poland; krzysztof.skrzypiec@mail.umcs.pl; 5 Department of Biophysics, Institute of Physics, Faculty of Mathematics, Physics and Informatics, Maria Curie-Skłodowska University, M.C. Skłodowska Square 1, 20-031 Lublin, Poland; wieslaw.gruszecki@mail.umcs.pl

**Keywords:** host defense peptides, antimicrobial peptides, proline-rich peptides, *Galleria mellonella*, atomic force microscopy, FTIR spectroscopy, SEM

## Abstract

Two proline-rich antimicrobial peptides (PrAMPs), named P1 and P2, purified from hemolymph of the greater wax moth *Galleria mellonella*, were studied for their effects on Gram-negative (*Escherichia coli*) and Gram-positive (*Micrococcus luteus*) bacteria. Both peptides decreased the *M. luteus* bacterial survival rate and caused *E. coli* bacterial membrane permeabilization. However, in both cases, the P2 peptide was approximately three times more effective than the P1 peptide. Fluorescence microscopy imaging demonstrated binding of both FITC-labeled peptides to *E. coli* and *M. luteus* cells. Atomic force microscopy (AFM) and scanning electron microscopy (SEM) imaging of peptide-treated bacteria revealed considerable changes in cell morphology, cell surface topography, and nanomechanical properties. The interactions of the PrAMPs with bacterial cells were also analyzed by FTIR spectroscopy. The P1 peptide action toward *E. coli* led to partial aggregation of proteins, whereas treatment with P2 resulted in reduced protein aggregation, reflecting differences between both *G. mellonella* PrAMPs antibacterial action. Moreover, both PrAMPs caused a decrease and an increase in the protein content in relation to lipids on the *E. coli* and *M. luteus* cell surface, respectively. The obtained results reflect not only differences between the *G. mellonella* P1 and P2 peptides but also differences in the cell surface between Gram-negative and Gram-positive bacteria. Both characterized *G. mellonella* PrAMPs are further representatives of proline-rich peptides with a membrane-permeabilizing antimicrobial mode of action.

## 1. Introduction

Antimicrobial peptides (AMPs) are key effector molecules of innate immunity in animals, including invertebrate and vertebrate species. Usually, they are small (3–10 kDa) and cationic molecules with an amphipathic character. Based on their structure, AMPs can be classified into three groups: (i) linear peptides without cysteine residues that can form α-helices, (ii) peptides with cysteine residues forming disulfide bridges responsible for stabilizing the spatial conformation, and (iii) peptides with overrepresentation of one of the amino acids, e.g., proline, glycine, tryptophan, or histidine [1,2]. Thanks to their basic character and the presence of amphipathic regions, AMPs can interact with microbial cell surface and phospholipid membranes, which are considered as main targets of their action. Binding of AMPs to such membranes can lead to depolarization and disruption of the proper membrane structure, eventually resulting in death of pathogen cells. There are several models describing the mechanisms of AMP interaction with phospholipid membranes, including the carpet model, barrel-stave channel formation, toroidal pore formation, the aggregate model, and others [3,4,5,6,7]. However, there are AMPs that can traverse through microbial cell membranes, enter the cytoplasm, and reach intracellular targets, e.g., nucleic acids, components of the translational machinery, and chaperons. Such interactions can disrupt fundamental life processes, i.e., replication, transcription, translation, and protein folding, eventually causing cell death [8,9]. Some examples of AMPs targeting intracellular structures and processes have been described among proline-rich antimicrobial peptides (PrAMPs) of insect origin, e.g., dipteran drosocin, hymenopteran abaecins and apidaecins, hemipteran pyrrhocoricin, and metalnikowin [10].

The greater wax moth *Galleria mellonella* has been widely used as a model organism in research on insect innate immunity mechanisms, host–pathogen interactions, and as an alternative model for in vivo tests of virulence factors of different pathogens and efficacy of new antimicrobial molecules and therapies [11,12,13,14,15]. In addition, *G. mellonella* is also a valuable in vivo model for investigating the toxicity, therapeutic potential, and immune interactions of different metal nanoparticles [16]. More than 20 different AMPs with diverse biochemical and antimicrobial properties have been reported in *G. mellonella* [17,18,19], and new peptides and proteins with antimicrobial activity are still being discovered in this species [20,21,22,23]. Among the rich repertoire of *G. mellonella* AMPs, two proline-rich peptides have been reported, namely proline-rich peptide 1 (P1; amino acid sequence: DIQIPGIKKPTHRDIIIPNWNPNVRTQPWQRFGGNKS; prolines count 13.5% of total amino acids number) and proline-rich peptide 2 (P2; amino acid sequence: EIRLPEPFRFPSPTVPKPIDIDPILPHPWSPRQTYPIIARRS; proline content 26.2%) [17]. These peptides are encoded by one gene (*gmpro*) containing sequences coding for P1, P2, anionic peptide 1, and heliocin-like peptide. Increased levels of their mRNAs were reported in the fat body and gut of *G. mellonella* larvae infected with *Pseudomonas entomophila*, an entomopathogenic Gram-negative bacterium [23,24]. The precursory GmPro polypeptide is 202 amino acid long and exhibits some similarities to lepidopteran lebocins; however, it does not contain a mature lebocin sequence. It was suggested that the GmPro and GmPro-derived peptides are specifically processed in the insect hemolymph [19]. Searching for sequence similarities of both *G. mellonella* PrAMPs revealed P1 peptide similarities mainly with peptide sequences of lepidopteran species (BLASTP server database release 2.16.0+ available at https://www.uniprot.org/blast/uniprotkb; accessed on 09 July 2025) [25,26]. Peptide sequences with relatively high similarity to the *G. mellonella* P1 peptide have been found in *Pararge aegeria* (Jg11823 protein; A0A8S4SE67), *Vanessa tameamea* (uncharacterized protein; A0A8B8IF95), *Danaus chrysippus* (hypothetical protein; A0A8J2WBB6), *Brenthis ino* (uncharacterized protein fragment; A0A8J9VL52), *Papilio xuthus* (lebocin-1/2; A0A194PKF6), *Papilio machon* (lebocin-1/2; A0A194QKS8), *Pieris macdunnoughi* (uncharacterized protein; A0A821 × 1G5), and *Pieris brassicae* (uncharacterized protein; A0A9P0XJI3). In contrast, the P2 peptide seems to be rather unique for *G. mellonella* because such similarities have not been found. Both PrAMPs exhibited antibacterial activity against the Gram-positive bacterium *Micrococcus luteus*, and the P1 peptide had antifungal activity against some yeasts and yeast-like fungi [17]. An increased level of both PrAMPs in the hemolymph was detected in *G. mellonella* larvae infected with different *Pseudomonas aeruginosa* strains [27] and *Aspergillus niger* α-1,3-glucan [28]. It was also reported that the P2 peptide level in *G. mellonella* hemolymph increased after challenge with Gram-negative (*Escherichia coli*) and Gram-positive (*M. luteus*) bacteria [29].

In this work, we investigated in more detail the interactions of both *G. mellonella* PrAMPs purified from larval hemolymph with Gram-negative (*E. coli*) and Gram-positive (*M. luteus*) bacteria by scanning electron microscopy (SEM), atomic force microscopy (AFM), and Fourier-transform infrared absorption spectroscopy (FTIR). In addition, a β-galactosidase assay was used to evaluate the ability of both PrAMPs to permeabilize bacterial membranes.

## 2. Results

### 2.1. Interaction of G. mellonella PrAMPs with Micrococcus luteus Cells

#### 2.1.1. Evaluation of Anti-*M. luteus* Activity

**Figure 1:**
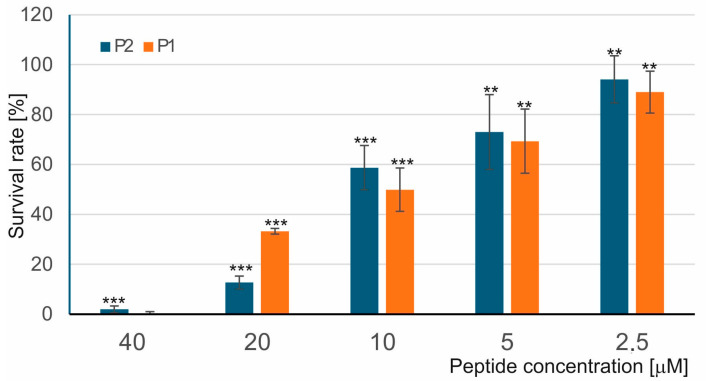
Antibacterial activity of the P1 and P2 peptides against *M. luteus*. The bacteria were incubated without and with the P1 and P2 peptides for 60 min, and then the survival rate was evaluated by LIVE/DEAD staining as described in Section 4. The survival rate of bacteria incubated without the peptides was considered as 100%. Statistical significances were marked with asterisks: ** *p* ≤ 0.01, *** *p* ≤ 0.001.

*Micrococcus luteus* cells were incubated with different concentrations of the P1 and P2 peptides (2.5–40 µM), and their survival was evaluated using a LIVE/DEAD assay. As presented in Figure 1, both peptides were able to kill bacterial cells in vitro, and their survival rate decreased gradually with the increasing peptide concentration. At the 10 µM concentration, the survival rate of *M. luteus* treated with the P1 and P2 peptides was calculated as approx. 50% and 60%, respectively. However, the P2 peptide was more effective than the P1 peptide at the 20 µM concentration, at which the survival rate of the bacteria treated with the P1 and P2 peptides was calculated as approx. 37% and 10%, respectively (Figure 1 and Figure S1). The increase in the number of propidium iodide (PI)-positive cells indicated the loss of bacterial membrane integrity and its damage occurring under the influence of both peptides.

#### 2.1.2. Morphology of *M. luteus* Cells After Treatment with P1 and P2 Peptides (SEM Imaging)

**Figure 2:**
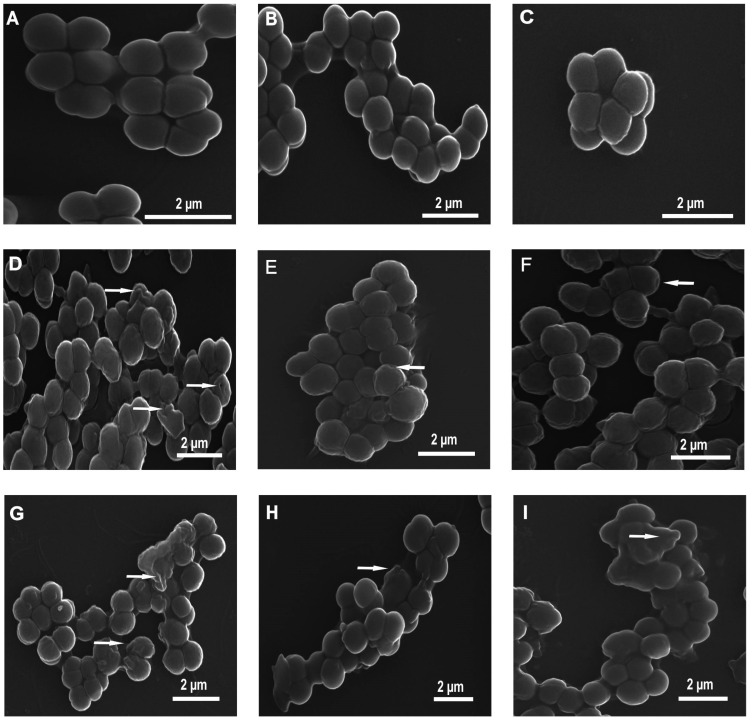
SEM micrographs of *M. luteus* cells after 60-min treatment with 10 µM P1 and P2 peptides. Control cells (**A**–**C**) and peptide-treated cells (**D**–**I**). (**A**–**C**) Clusters of control cells showing a well-preserved smooth and intact cell surface; (**D**–**F**) cells incubated with the P1 peptide showing different changes: small indentations and corrugated surfaces; (**G**–**I**) bacteria with visible different alterations in the cell surface after exposure to P2 peptide; indentations and bulges with different shapes. The alterations are indicated by the arrows.

**Figure 3:**
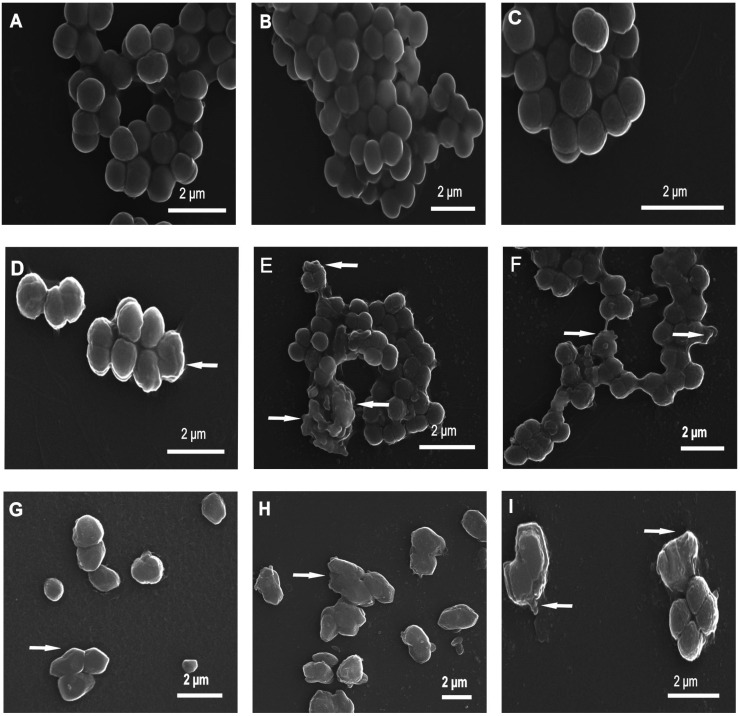
SEM micrographs of *M. luteus* cells after 90-min treatment with 10 µM P1 and P2 peptides. Cells from control samples (**A**–**C**) and peptide-treated cells (**D**–**I**). (**A**–**C**) The control cells are spherical and intact; (**D**–**F**) cells treated with the P1 peptide showing no smooth surface with visible small round bubbles and irregular protrusions; (**G**–**I**) cells incubated with the P2 peptide displaying some indentations and irregular protrusions. The alterations are indicated by the arrows.

The changes in the *M. luteus* cell morphology were imaged by scanning electron microscopy (SEM) after the 60- and 90-min treatment with the P1 and P2 peptides (Figure 2 and Figure 3). The control *M. luteus* cells showed a smooth and intact surface and had a typical spherical shape. The cells were grouped predominantly in tetrads and in bigger clusters (Figure 2A–C and Figure 3A–C).

After the 60-min incubation with the P1 and P2 peptides, the cells displayed changed appearance (Figure 2). The cells treated with the P1 peptide had corrugated surfaces. They were grouped in big clusters and exhibited some small indentations (Figure 2D–F). After 60 min of treatment with the P2 peptide, indentations in the surfaces along with bulges of different shapes were observed (Figure 2G–I).

After the 90-min incubation with the P1 and P2 peptides, altered cells were also observed in comparison to the control cells (Figure 3). The surface of the cells incubated with the P1 peptide was not smooth but exhibited small round bubbles and irregular protrusions (Figure 3D–F). The P2 peptide also affected the surface of the cells. As presented in Figure 3, their surface exhibited indentations and irregular protrusions as well (Figure 3G–I).

#### 2.1.3. *M. luteus* Cell Surface Topography and Nanomechanical Properties After Treatment with P1 and P2 Peptides (AFM Imaging)

**Figure 4:**
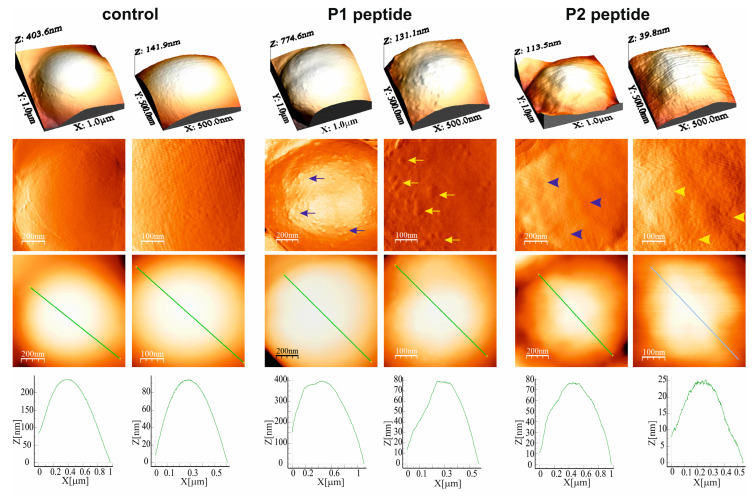
AFM imaging of *M. luteus* cells after 60-min treatment with 5 µM P1 and P2 peptides. 3D images, topography (Peak Force Error) images, height images, and section profiles are presented (1 µm × 1 µm and 500 nm × 500 nm). The arrows and arrowheads indicate numerous small lumps and a shallowly wavy cell surface, respectively.

The treatment of *M. luteus* with both PrAMPs resulted in alterations in the cell surface topography, as presented in the different type AFM images (Figure 4). The Peak Force Error images clearly evidenced the presence of numerous small lumps on the cell surface after the P1 peptide treatment, whereas after P2 peptide treatment the cell surface was shallowly wavy, which was not observed on the surface of the control cells. As presented in the section profiles, the exposure of the *M. luteus* cells to the P2 peptide also caused a considerable decrease in the cell height from approx. 400 nm to approx. 115 nm (Figure 4). These alterations in cell surface topography were accompanied by changes in the nanomechanical properties of the cell surface (Table S1). The P1 and P2 peptide-treated *M. luteus* cells had significantly decreased RMS roughness and Young’s modulus values. In turn, the adhesion forces were significantly reduced after the 60-min incubation with the P1 peptide but increased after the treatment with the P2 peptide (Table S1).

### 2.2. Interaction of G. mellonella PrAMPs with Escherichia coli Cells

#### 2.2.1. Permeabilization of Bacterial Membrane by P1 and P2 Peptides

**Figure 5:**
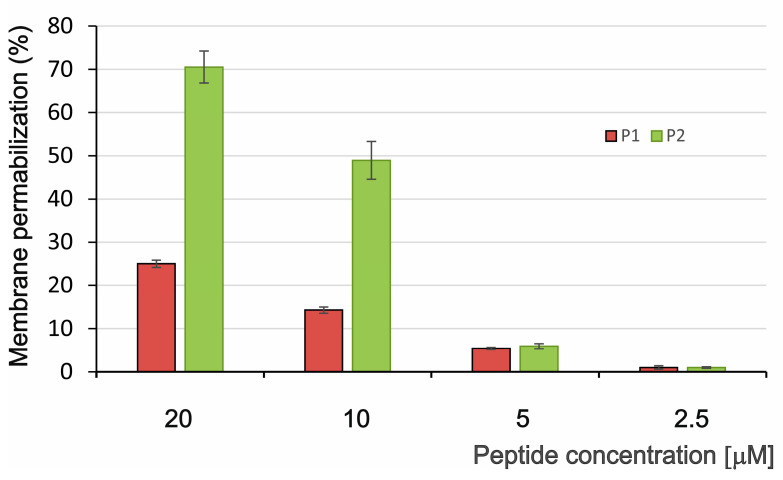
*E. coli* membrane permeabilization by the P1 and P2 peptides. The bacteria were incubated without and with different concentrations of the peptides for 60 min, and then the β-galactosidase assay was performed. The permeabilization level was calculated as described in Section 4.

In our previous studies, both *G. mellonella* PrAMPs did not inhibit the growth of Gram-negative bacteria tested, i.e., *E. coli* D31, *E. coli* ATCC 25922, and *Salmonella typhimurium* [17]. Nevertheless, we decided to verify these results by performing permeabilization tests using a β-galactosidase assay with another *E. coli* strain, i.e., *E. coli* JM83. As demonstrated in Figure 5, the P1 peptide permeabilized the bacterial membrane weakly during 60-min incubation, showing approx. 15% and 25% membrane permeabilization at the 10 µM and 20 µM concentration, respectively (Figure 5). In contrast, the P2 peptide was much more membrane active showing a permeabilization level of approx. 50% and 70% at the 10 µM and 20 µM concentration, respectively (Figure 5).

#### 2.2.2. Morphology of *E. coli* Cells After Treatment with P1 and P2 Peptides (SEM Imaging)

**Figure 6:**
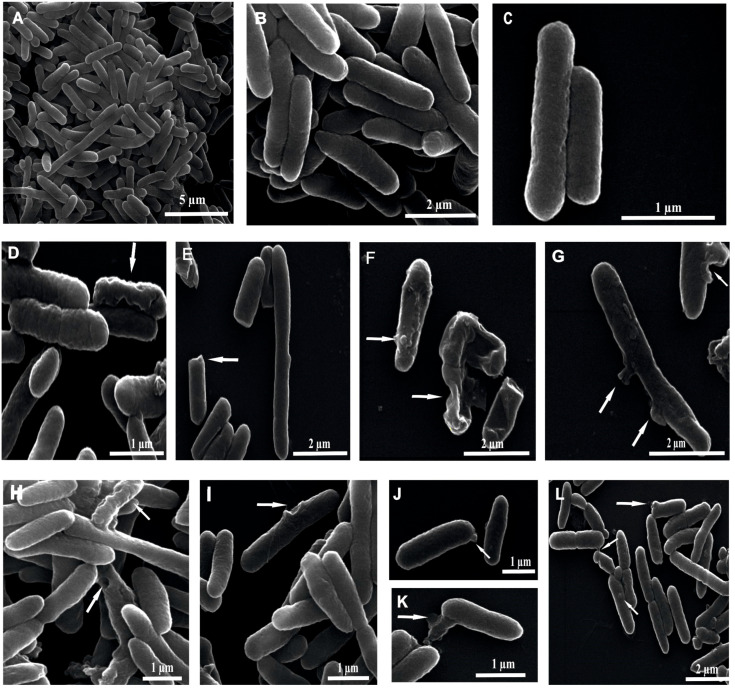
SEM micrographs of *E. coli* cells after 45-min treatment with 10 µM P1 and P2 peptides. Control cells (**A**–**C**) and peptide-treated cells (**D**–**L**). (**A**) control cells showing a well-preserved cell surface; (**B**,**C**) enlarged view of control cells showing a smooth and intact cell surface; (**D**–**G**) cells incubated with the P1 peptide showing different changes: dimples, protrusions, surface corrugation, and bulges; (**H**–**L**) bacteria after exposure to the P2 peptide with visible different alterations in the cell surface: indentations, bubbles, and bulges with different shapes. The alterations are indicated by the arrows.

**Figure 7:**
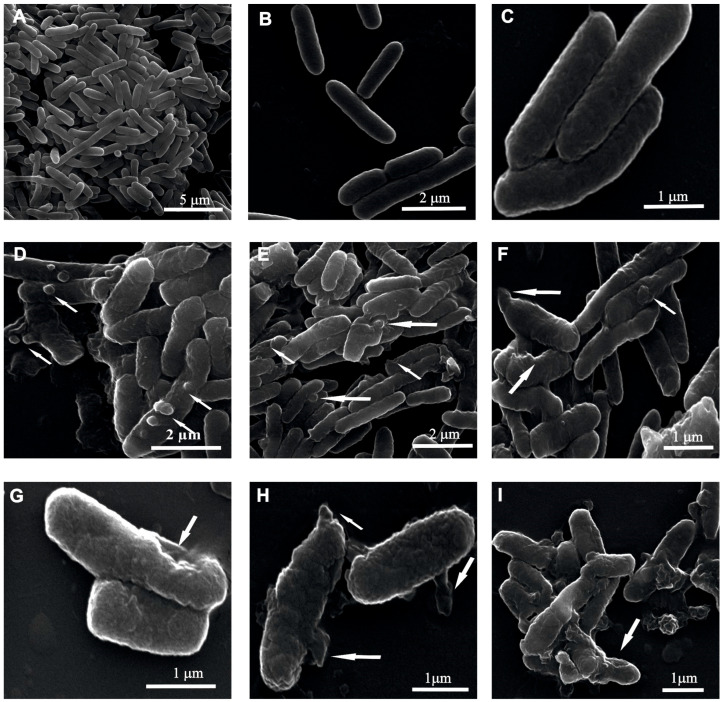
SEM micrographs of *E. coli* treated with 10 µM P1 and P2 peptides for 90 min. Cells from control samples (**A**–**C**) and peptide-treated cells (**D**–**I**). (**A**–**C**) The control cells are long and intact; (**D**–**F**) cells treated with the P1 peptide showing round bubbles, dimples, and irregular protrusions; (**G**–**I**) cells incubated with the P2 peptide displaying wide pits, irregular protrusions, and a strongly changed shape. The alterations are indicated by the arrows.

The changes in the *E. coli* cell morphology were imaged by scanning electron microscopy (SEM) after 45- and 90-min treatment with the P1 and P2 peptides at the 10 µM concentration (Figure 6 and Figure 7). The untreated *E. coli* cells were rod-shaped and about 2–3.5 µm long. The undamaged control cells displayed a smooth and intact surface (Figure 6A–C and Figure 7A–C).

After incubation with the P1 and P2 peptides for 45 min, the cells displayed changed appearance (Figure 6D–L). The surface of some cells treated with the examined peptides looked corrugated. There were also some dimples in the cells (Figure 6D,G–I). Along with the dimples on the surface, protrusions with various shapes and sizes were found in some cells. These protruding fragments sometimes resembled bubbles or blisters (Figure 6F,J,L). Bigger protrusions with an undefined shape were seen in other cells (Figure 6G,K).

After the 90-min incubation with the P1 and P2 peptides, cells with an altered shape were observed, in comparison to the control cells (Figure 7D–I). In some cells, the extent of the damage and change in the surface was very huge (Figure 7I). One of the alterations noticed in the P1 peptide-treated cells was the formation of many small, roughly similar, round shaped bubbles on the cell surface (Figure 7D). Along with the corrugated surface, small dimples were noted and bubbles in some cells (Figure 7E,F). Among the bacterial cells, especially those treated with the P2 peptide, strongly deformed cells with irregular protrusions and bulges with various shapes were seen (Figure 7G,H). In some cells, deep and wide pits were also found (Figure 7G).

#### 2.2.3. *E. coli* Cell Surface Topography and Nanomechanical Properties After Treatment with P1 and P2 Peptides (AFM Imaging)

**Figure 8:**
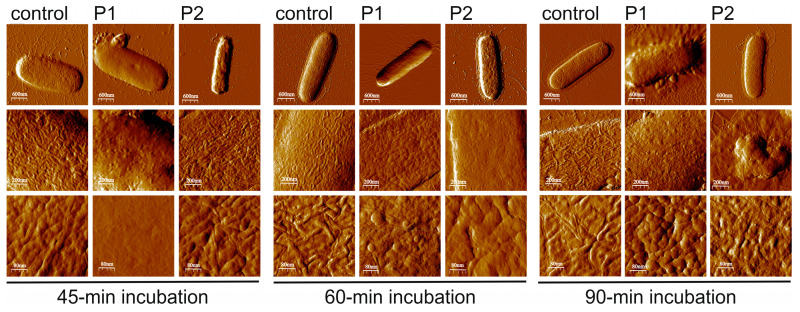
AFM imaging of *E. coli* cells after treatment with the P1 and P2 peptides—surface topography. The bacteria were incubated without and with the peptides at the 10 µM concentration for 45–90 min. The Peak Force Error images are presented.

**Figure 9:**
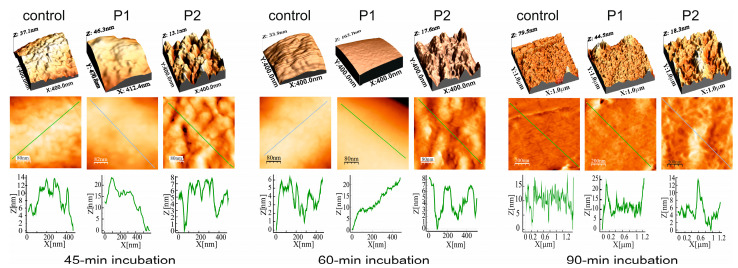
AFM imaging of *E. coli* cells after treatment with the P1 and P2 peptides—section profiles. The bacteria were incubated without and with the peptides at the 10 µM concentration for 45–90 min. The 3D images and section profiles are presented. The green and blue lines in the height images indicated section profiles.

The treatment of the *E. coli* cells with both PrAMPs resulted in alterations in the cell surface topography, as presented in the Peak Force Error AFM images (Figure 8) and the 3D images and section profiles (Figure 9). However, the changes in the cell surface topography and in the measured nanomechanical properties were clearly dependent on the peptide. The Peak Force Error and 3D images revealed that the *E. coli* treatment with the P1 peptide for 45 and 60 min caused smoothing of the cell surface, while at the same time, the treatment with the P2 peptide resulted in the appearance of clear unevenness on the surface. The alterations caused by the P2 peptide were well reflected by the significantly increased RMS roughness values measured at both incubation times and by the increased adhesion forces and decreased Young’s modulus calculated for the 45-min incubation (Table S2). In the case of the *E. coli* exposure to the P1 peptide, the adhesion forces were significantly decreased after the 60- and 90-min incubation, while the Young’s modulus was temporarily decreased after the 60-min incubation and then increased in comparison with the control cells (Table S2).

### 2.3. Binding of P1 and P2 Peptides to M. luteus and E. coli Cells

The effects of the action of both *G. mellonella* PrAMPs against *M. luteus* and *E. coli* suggested that the peptides were able to bind to the bacterial cells. Binding of both peptides to *M. luteus* and *E. coli* cells was demonstrated by laser scanning confocal microscopy (LSCM) imaging after incubation of the bacteria with the FITC-labeled P1 and P2 peptides (Figure S2).

### 2.4. FTIR Spectroscopy Investigation of P1 and P2 Effects on M. luteus and E. coli Cells

The action of the P1 and P2 peptides against *M. luteus* and *E. coli* cells was examined with the Fourier-transform infrared absorption spectroscopy (FTIR) technique. The characteristic vibrational bands of the peptides are not shown due to the low concentration of these compounds relative to cellular components. The spectra were normalized by dividing by surface area beneath the bands from the 3600–970 cm^−1^ region.

#### 2.4.1. Effects on *E. coli* Cells

**Figure 10:**
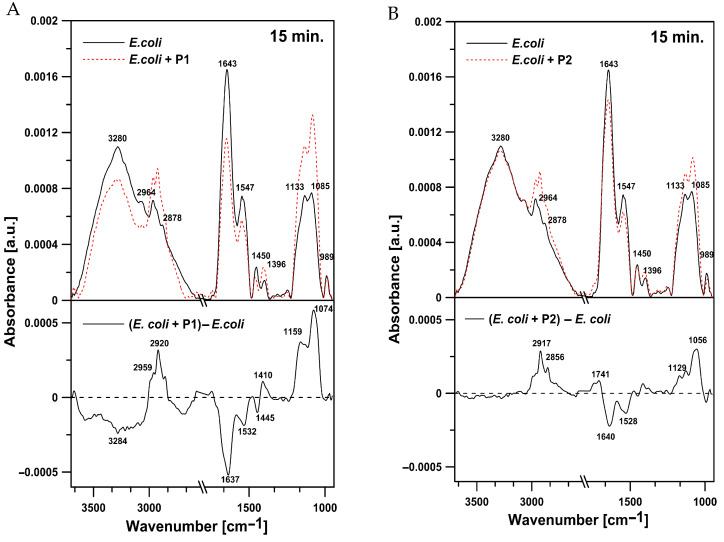
(**A**) ATR-FTIR spectra of control *E. coli* cells (upper panel, black solid line), *E. coli* cells incubated with the P1 peptide (upper panel, red dotted line), and the difference spectrum (lower panel). (**B**) ATR-FTIR spectra of *E. coli* cells (upper panel, black solid line), *E. coli* cells incubated with the P2 peptide (upper panel, red dotted line), and the difference spectrum (lower panel).

Representative IR spectra of *E. coli* cells in the region between 3600 and 970 cm^−1^ after the 15-min treatment with the tested peptides are presented in Figure 10. Incorporated into the *E. coli* cells, the P1 and P2 peptides exerted effects in the 3300–3100 cm^−1^ (amide A), 3000–2800 cm^−1^ (lipids), 1700–1600 cm^−1^ (amide I), 1580–1510 cm^−1^ (amide II), and 1200–900 cm^−1^ spectral regions. In *E. coli* cells exposed to the tested peptides, compared to the control cells, significant changes in the overall intensity of all the peaks were noted (Figure 10). After the treatment with the P1 peptide, an increase in oscillator strength was observed in the bands representing symmetric (2850 cm^−1^) and antisymmetric (2920 cm^−1^) stretching vibrations of the CH_2_ and CH_3_ groups of alkyl chains. Moreover, a rise in the intensity of the band centered at 1085 cm^−1^, characteristic for symmetric stretching vibrations of the PO_2_^−^ groups, was revealed. On the other hand, the analysis of the spectra of *E. coli* cells incubated with P1 peptide showed a significant decrease in the amide I (1643 cm^−1^) and amide II (1547 cm^−1^) intensity. The presence of a negative band in this region indicates a reduction in the relative content of protein in relation to lipids (Figure 10A). Similar changes were observed in the cells incubated with the P2 peptide. Simultaneously, the treatment with the P2 peptide increased the intensity of the band with the maximum at 1741 cm^−1^ assigned to the ester carbonyl groups C=O of phospholipids (Figure 10B)

**Figure 11:**
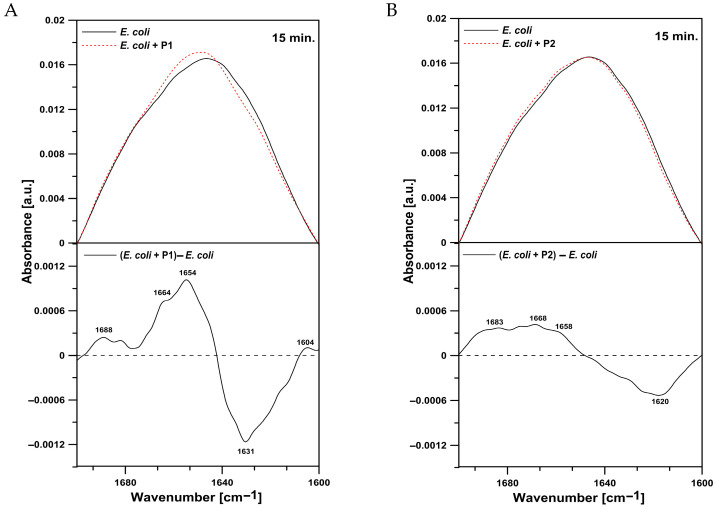
(**A**) FTIR spectrum from the amide I region in *E. coli* cells (upper panel, black solid line), cells treated with the P1 peptide (upper panel, red dotted line), and the difference spectrum (lower panel). (**B**) FTIR spectrum from the amide I region in *E. coli* cells (upper panel, black solid line), cells treated with the P2 peptide (upper panel, red dotted line), and the difference spectrum (lower panel).

**Figure 12:**
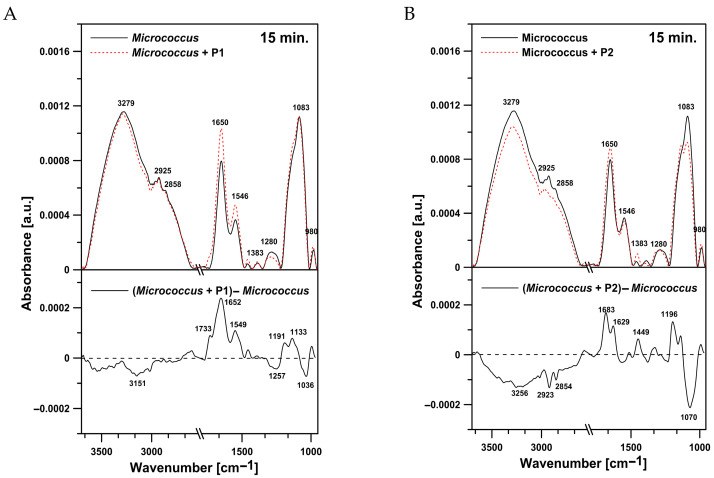
(**A**) ATR-FTIR spectra of control *M. luteus* cells (upper panel, black solid line), *M. luteus* cells incubated with the P1 peptide (upper panel, red dotted line), and the difference spectrum (lower panel). (**B**) ATR-FTIR spectra of control *M. luteus* cells (upper panel, black solid line), *M. luteus* cells incubated with the P2 peptide (upper panel, red dotted line), and the difference spectrum (lower panel).

In the current study, the amide I region representing the overall secondary structure and molecular organization of proteins was analyzed (Figure 11). The peaks in the region of amide I were assigned to antiparallel β-sheets (1675–1695 cm^−1^), aggregated strands (1610–1628 cm^−1^), α-helices (1648–1660 cm^−1^), β-sheets (1625–1640 cm^−1^), unordered structures (1652–1660 cm^−1^), and turns (1660–1685 cm^−1^) [30]. The analysis of this region revealed that the addition of the P1 and P2 peptides caused an increase in antiparallel β-sheets (1688 cm^−1^ and 1683 cm^−1^), turns (1664 cm^−1^ and 1668 cm^−1^), and unordered structures (1654 cm^−1^ and 1658 cm^−1^) and a decrease in β-sheets (1631 cm^−1^) (Figure 11A,B). Furthermore, the increase at 1688 cm^−1^ observed in cells treated with the P1 peptide can be also assigned to an increase in aggregated structures (Figure 11A). In the P2 peptide-treated bacteria, a decrease in aggregated strands (1620 cm^−1^) was noticed. Such a result suggests partial aggregation of different proteins in *E. coli* cells after the treatment with both P1 and P2 peptides; however, the changes were more pronounced after the incubation with the P1 peptide (Figure 12).

#### 2.4.2. Effects on *M. luteus* Cells

**Figure 13:**
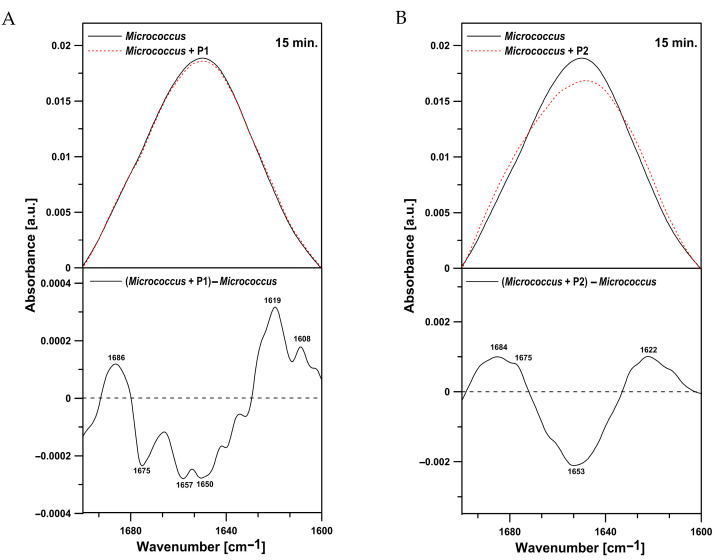
(**A**) FTIR spectrum from the amide I region in *M. luteus* cells (upper panel, black solid line), cells treated with the P1 peptide (upper panel, red dotted line), and the difference spectrum (lower panel). (**B**) FTIR spectrum from the amide I region in *M. luteus* cells (upper panel, black solid line), cells treated with the P2 peptide (upper panel, red dotted line), and the difference spectrum (lower panel).

Different results were obtained for the *M. luteus* cells. The bacteria treated with the P1 peptide (Figure 12A) exhibited a clear increase in protein content in relation to lipids. Furthermore, a positive peak with a maximum at 1733 cm^−1^, corresponding to the ester carbonyl groups of phospholipids, was noted in the difference spectrum. In the presence of the P2 peptide, a pronounced effect associated with an increase in the oscillator strength of the band characteristic for vibrations of amide I was revealed (Figure 12B). At the same time, the P2 treatment resulted in reduced intensity of antisymmetric (2923 cm^−1^) and symmetric (2854 cm^−1^) stretching vibrations in the CH_2_ groups of alkyl chains. Changes related to the presence of the P2 peptide were also observed in the region between 1200 and 1070 cm^−1^, usually attributed to phosphate associated with nucleic acids and phospholipids. A drop in the oscillator strength in the region corresponding to C-O-P-O-C symmetric stretching vibrations (1070 cm^−1^) was noted (Figure 12B). The increase in the intensity of the amide I band probably indicates that additional proteins could be exposed on the surface of *M. luteus* cells incubated with the peptides. The analysis of the region between 1700 and 1600 cm^−1^ of *M. luteus* cells after the treatment with the P1 and P2 peptides showed an increase in antiparallel β-sheets and aggregated strands with a simultaneous decrease in α-helices (Figure 13A,B).

## 3. Discussion

Proline-rich antimicrobial peptides constitute a family of peptides that vary in their length and the mode of antimicrobial action. In insects, short- and long-chain PrAMPs can be distinguished [31,32]. Short-chain PrAMPs are composed of less than 20 amino acid residues, and most of them are more active against Gram-negative than Gram-positive bacteria. This subfamily is represented by e.g., apidaecins, drosocin, formecins, metalnikowins, and pyrrhocoricin. Long-chain PrAMPs contain more than 20 amino acid residues and can be active against both Gram-negative and Gram-positive bacteria. They include abaecins, lebocins, and metchnikowin. In different natural PrAMPs, proline residues can be organized in Pro-Arg-Pro (PRP) or Pro-His-Pro (PHP) motifs [10,32,33].

According to a recently proposed definition, a PrAMP should meet the following criteria: (i) proline content in a peptide chain more than 25%, (ii) modulation of DnaK and/or the 70S ribosome, (iii) net charge at least +1, and (iv) synthesis in response to bacterial infection and/or with pronounced antimicrobial activity [34]. However, among the naturally occurring AMPs described so far, there are some molecules classified as PrAMPs that do not meet all the recently proposed characteristics, e.g., the *G. mellonella* P1 and P2 peptides described in this work.

As demonstrated in this study, the *G. mellonella* P1 and P2 peptides exhibited activity against Gram-positive (*M. luteus*) and Gram-negative (*E. coli* JM83) bacteria and affected the integrity of bacterial membranes. In our previous paper, it was shown that among the tested Gram-positive bacteria, only *M. luteus* was moderately susceptible to both *G. mellonella* PrAMPs, while the tested Gram-negative bacteria were not killed by these peptides [17]. Hence, our previous results were only partly confirmed in our current work. These discrepancies could be explained by the fact that different methods (optical density measurements *versus* LIVE/DEAD staining and β-galactosidase assay) and Gram-negative bacterial strains (*E. coli* D31, *E. coli* ATCC 25,922 *versus E. coli* JM83) were used.

Binding of the P1 and P2 peptides to *M. luteus* and *E. coli* cells was demonstrated in this study using fluorescently labeled peptides and LSCM imaging. Further studies are needed to determine whether the peptides bind to the bacterial cell surface or bacterial cell membranes or whether they are able to traverse the membranes and enter the cells. Considering the mode of antimicrobial action, PrAMPs can be divided into non-lytic and lytic peptides. It has been well documented that in Gram-negative bacteria, some PrAMPs, including the insect ones, have intracellular targets, such as DnaK chaperones and/or 70S ribosomes [10,35,36]. These peptides kill bacteria via a non-lytic mechanism and without damaging the bacterial envelope. However, non-lytic PrAMPs require inner membrane transporters, e.g., SbmA/BacA [37] or pore-forming AMPs [38,39] to be internalized into the bacterial cell. PrAMPs using the non-lytic mode of action contain regions with consensus sequences needed for interaction with DnaK and/or with 70S ribosomes. An analysis of amino acid sequences of both *G. mellonella* PrAMPs aimed at prediction of sequences involved in DnaK binding (the limbo server available at http://limbo.switchlab.org; accessed on 24 September 2024) [39,40] indicated that only the P2 peptide would interact with this chaperone through a non-canonical sequence comprising the first seven amino acids at the N-terminus, i.e., EIRLPEP (score 12.0; option used: best overall prediction—heptapeptides with scores above the value of 11.08, are predicted DnaK binders). The consensus amino acid sequence XXR/YLPRPRX has been shown to be essential for binding in the ribosomal exit tunnel and thus, for the antibacterial activity of non-lytic PrAMPs [41]; however, it is not present in the peptide chains of the *G. mellonella* PrAMPs, suggesting another mode of antimicrobial action. Our results demonstrated permeabilization of the *E. coli* membrane (β-galactosidase assay) as well as penetration of membrane-impermeable propidium iodide into *M. luteus* cells (LIVE/DEAD staining) after the treatment with the P1 and P2 peptides, although to a different extent. This indicates that both peptides can permeabilize membranes of Gram-negative and Gram-positive bacteria, suggesting a membrane-targeting mode of action. Nevertheless, it cannot be excluded that, after reaching the bacterial intracellular space, the P2 peptide can interact with DnaK. PrAMPs with bacterial membrane permeabilizing activity, including those with a dual mode of action, have been described in different animals, e.g., *Mytilus galloprovincialis* myticalins [42], crustacean penaeidins, crustins, astacidins, and arasins [43,44,45,46,47], some insect lebocins [48,49], and cetacean PrAMPs [50]. Interestingly, by mining genomes of different *Camelidae* species, in the alpaca *Vicugna pacos*, Panteleev et al. [51] found a PrAMP of Bac7-like family in which N-terminal region was responsible for the inhibition of bacterial protein synthesis in vitro, whereas the C-terminal region allowed the peptide to penetrate bacterial membranes [51]. Other mammalian PrAMP, i.e., Bac7(1-35), was demonstrated to kill *Pseudomonas aeruginosa* cells, including multidrug-resistant cystic fibrosis isolates, by membrane damage resulting in membranolytic effects [52]. Considering that Bac7(1-35) can kill *E. coli* cells by targeting bacterial ribosome and inhibiting protein synthesis, the report also indicated that Bac7 can kill bacterial cells by variable modes of action that depend on the characteristics of the target cells [52].

The different effects of the P1 and P2 peptides on bacterial cell morphology (SEM), cell surface topography, and nanomechanical properties (AFM) clearly depended on the bacterial strain and the characteristics of the peptide. In the case of *E. coli*, both peptides exhibited different capabilities of bacterial membrane permeabilization, with the P2 peptide being approximately three times more membrane active than the P1 peptide (at the 10 µM and 20 µM concentrations). On the other hand, in *M. luteus* both PrAMPs affected bacterial membrane integrity and decreased survival rate to a similar extent at the 2.5–10 µM concentrations; however, at the 20 µM concentration, the P2 peptide was also approximately three times more effective than the P1 peptide in reducing the bacterial survival rate. These results indicate that the P2 peptide is more active than the P1 peptide against both the Gram-negative and Gram-positive bacteria tested. Interestingly, it was demonstrated that the *G. mellonella* P1 peptide, in contrast to the P2 peptide, also has antifungal activity against yeast and yeast-like fungi [17]. It cannot be excluded that both PrAMPs play different functions in *G. mellonella*, with the P1 and P2 peptides being mainly antifungal and antibacterial factors, respectively.

When the interactions of both PrAMPs with bacterial cells were analyzed by FTIR spectroscopy, it was revealed that both peptides caused a decrease and an increase in the protein content in relation to lipids in the *E. coli* and *M. luteus* cells, respectively. This result can reflect the well-known differences in the cell surface structure and cell envelope composition between Gram-negative and Gram-positive bacteria [53,54,55,56], which together with the peptide properties, influence the interactions with different AMPs [57,58]. In addition, the P1 peptide interaction with *E. coli* led to partial aggregation of proteins, whereas the P2 treatment resulted in a decrease in protein aggregation, reflecting probably the sequence and conformation differences between both *G. mellonella* PrAMPs. Such differences were not noticed when *M. luteus* was incubated with the P1 and P2 peptides. The obtained results reflect not only differences between the *G. mellonella* P1 and P2 peptides but also between the cell surface of Gram-negative and Gram-positive bacteria.

The results presented in this study indicate that the antibacterial activity of both *G. mellonella* proline-rich peptides is connected with permeabilization of bacterial membrane leading to evident alterations in cell morphology (SEM), cell surface topography, and nanomechanical properties (AFM), a shift in protein to lipid content, and changes in protein conformation (FTIR spectroscopy). Both *G. mellonella* PrAMPs are further representatives of proline-rich peptides with a membrane-permeabilizing antimicrobial mode of action.

## 4. Materials and Methods

### 4.1. Insect Rearing, Immunization, Hemolymph Collection, and Preparing of Methanolic Extracts

Last instar larvae (250–300 mg weight) of the greater wax moth *G. mellonella* (Lepidoptera: Pyralidae) from a continuous laboratory culture were used in the study. They were reared on honeybee nest debris and maintained at 30 °C and 70–80% humidity in the dark. For immunization of the larvae, a mixture of live *E. coli* D31 and *M. luteus* ATCC 10,240 bacteria and the pricking method was used. The immune hemolymph was collected 24 h post-treatment as described in our previous papers [17,29]. The hemocyte-free hemolymph was subjected to methanolic-acidic extraction, and the obtained extracts were delipidated, lyophilized, and stored at −80 °C until use [29].

### 4.2. Purification of Pro-Rich Peptides from Hemolymph of Immunized G. mellonella Larvae

**Figure 14:**
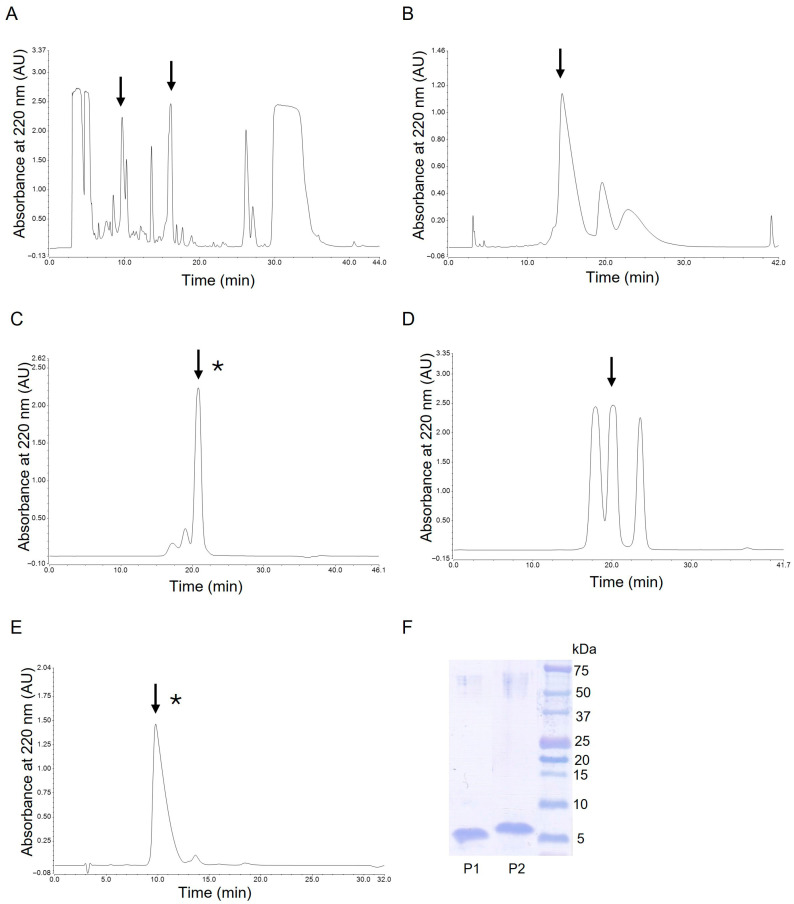
Chromatograms illustrating the purification steps of P1 and P2 peptides (panels (**A**–**E**)) and an SDS-PAGE image of the final preparations (panel (**F**)). The arrows indicate collected fractions, while the asterisks show peaks of purified homogenous peptides. The details of the purification steps are described in the Section 4.

P1 and P2 peptides were purified from the methanolic extract of *G. mellonella* immune hemolymph using a modified technique described in our previous study [17,29]. Briefly, the hemolymph extract was dissolved in 0.1% (*v*/*v*) trifluoroacetic acid (TFA) and subjected to reversed-phase high-pressure liquid chromatography (RP-HPLC) using a Discovery Bio Wide Pore C18 4.6 mm × 250 mm column (Sigma-Aldrich, St. Louis, MI, USA). Two buffers, A, 0.1% TFA (*v*/*v*) and B, 0.07% TFA, 80% acetonitrile (*v*/*v*), a linear gradient from 30 to 70% of buffer B over 35 min, and a 1 mL/min flow rate, were used. The spectrophotometric detection was conducted at 220 nm. The fractions eluting as two separate peaks in the range of 9.1–10.7 and 15.4–16.7 min (Figure 14A), containing P1 and P2 peptides, respectively, were collected, evaporated in a vacuum centrifuge, and subjected to further purification steps.

The fraction containing the P1 peptide was successively separated by RP-HPLC using the column, buffers, and flow rate as above, and a linear gradient from 30 to 32% of buffer B over 35 min. The fraction eluting at 12.7–17.3 min (Figure 14B) was collected, evaporated, and finally purified by gel filtration using a Superdex Peptide 10/300 GL column (Cytiva, Marlborough, MA, USA) in the isocratic gradient of 30% of buffer B at the 0.5 mL/min flow rate. The peak eluting at 20.8 min (Figure 14C), containing the homogenous P1 peptide, was collected and evaporated to dryness.

The fraction containing the P2 peptide was separated by gel filtration using a Superdex Peptide 10/300 GL column in the isocratic gradient of 30% buffer B at the 0.5 mL/min flow rate. The peak eluting at 19.2–21.2 min was collected, evaporated, and finally purified by RP-HPLC using a Supelcosil LC-18-DB 4.6 mm × 250 mm column (Supelco, Bellefonte, PA, USA) and a linear gradient from 38 to 58% of buffer B during 25 min. The broad peak containing the homogenous P2 peptide and eluting at 9.8 min was collected and evaporated to dryness.

All these chromatographic separations were performed at room temperature. The quantity of the obtained peptides was determined by amino acid analysis as described elsewhere [59], while the homogeneity and identity of the peptides was confirmed by SDS-PAGE electrophoresis (Figure 14F) [60] and by N-terminal sequencing using an automatic protein sequencer (Procise 491, Applied Biosystems, Foster City, CA, USA).

### 4.3. Fluorescent Labeling of G. mellonella Pro-Rich Peptides

Six nanomole portions of the P1 and P2 peptides were dissolved in 200 μL of sodium borate pH 8.5, and 10.5 μL of a dimethylsulfoxide solution containing 600 nanomoles of fluorescein isothiocyanate (FITC, isomer 1, Sigma-Aldrich, St. Louis, MI, USA) were added. The solution was mixed, incubated in darkness for 20 h at room temperature, and then the unreacted FITC was quenched by addition of 50 μL of a 1 M glycine solution in the borate buffer as above. After successive 30 min in darkness, the mixture was subjected to desalting using gel filtration chromatography on a Superdex Peptide 10/300 GL column, the isocratic gradient of 30% of buffer B, and the 0.5 mL/min flow rate. The peaks eluting at 17.5 and 16.0 min, containing FITC-P1 and FITC-P2 peptides, respectively, were collected and evaporated to dryness.

### 4.4. Binding of G. mellonella PrAMPs to Bacterial Cells

Log-phase *E. coli* JM83 and *M. luteus* ATCC 10,240 cells (60 μL of suspensions in 20 mM phosphate buffer, pH 7.4, OD_600_ = 0.2) were incubated with 10 μM of the FITC-P1 and FITC-P2 peptides at 37 °C (*E. coli*) and 30 °C (*M. luteus*) for 15 min. Then, the suspensions were centrifuged (4000× *g*, 10 min, 4 °C) and washed twice with 100 μL of 20 mM phosphate buffer, pH 7.4, containing 0.9% NaCl. Finally, the bacteria were suspended in 10 μL of 20 mM phosphate buffer, pH 7.4, and imaged using an LSM 5 PASCAL laser confocal scanning microscope (Carl Zeiss Microscopy, Jena, Germany) (excitation time: 600 ms; excitation and emission wavelengths: 470 and 520 nm, respectively) [61,62].

### 4.5. Bacterial Membrane Permeabilization—β-Galactosidase Assay

The permeabilization assay was performed using a suspension of mid-logarithmic phase *E. coli* JM83 cells (a strain bearing a pCH110 plasmid encoding constitutively synthesized cytoplasmic β-galactosidase) prepared in 20 mM phosphate buffer, pH 6.8, as described previously [62,63]. Briefly, the bacteria (5 × 10^5^ colony forming units, CFU) were added to the pre-incubated (15 min, 37 °C) peptide solutions prepared in 20 mM phosphate buffer, pH 6.8. The resulting suspensions were further incubated at 37 °C for 5–60 min (final concentrations of the peptides: 2.5–20.0 μM). Then, 20 mM HEPES/150 mM NaCl buffer, pH 7.5, and 50 mM *p*-nitrophenyl-β-D-galactopyranoside were added, and after 1.5-h incubation at 37 °C absorbance of the samples was measured at 405 nm using a multi-well plate spectrophotometer (Benchmark Plus, Bio-Rad, Hercules, CA, USA).

Live bacteria incubated without the peptides and bacteria permeabilized completely by 5 μM cecropin B (Sigma-Aldrich, USA) were used as controls. At the given time point, the percent of permeabilization was calculated assuming the permeabilization level of the cecropin B-treated bacteria as 100%, after subtracting the permeabilization level measured for the live bacteria. Three independent experiments with three technical repetitions for each sample type were performed.

### 4.6. Antibacterial Activity Against Micrococcus luteus

Anti-*M. luteus* (strain ATCC 10240) activity of both PrAMPs was tested using LIVE/DEAD staining essentially as described previously [64]. Briefly, log-phase bacterial cells in LB medium (10 μL of suspension, OD_600_ = 0.02) were incubated for 1 h without and with P1 and P2 peptides (final concentrations of the peptides: 2.5–40.0 μM) at 30 °C. After centrifugation (12,000× *g*, 10 min, 4 °C), the bacteria suspended in 0.85% NaCl were incubated for 1 h at room temperature. Finally, a LIVE/DEAD staining solution was added according to the manufacturer’s instruction (LIVE/DEAD BacLight Bacterial Viability Kit, Invitrogen, Carlsbad, CA, USA), and after 15-min incubation in darkness, the bacteria were observed using an LSM 5 PASCAL laser scanning confocal microscope (Carl Zeiss Microscopy, Jena, Germany).

### 4.7. Scanning Electron Microscopy (SEM) Imaging of Bacteria

Bacteria in the mid-exponential growth phase suspended in LB medium (OD_600_ = 0.2) were treated with the P1 and P2 peptides (final concentration: 10 µM) for 45 and 90 min at 37 °C (*E. coli*) and for 60 and 90 min at 30 °C (*M. luteus*). The bacteria were then fixed in 4% glutaraldehyde for 2 h. After rinsing several times with 0.1 M PBS, the cell pellets were post-fixed in a 1% (*v*/*v*) osmium tetroxide solution in 0.1 M phosphate buffer, pH 7.4, for 1 h at 4 °C. Then, the cells were dehydrated in a graded series of alcohol, dried in critical point, and coated with gold in an Emitech K550X Sputter Coater (Ashford, UK). The samples were observed with a TESCAN Vega 3 LMU microscope (Brno, Czech Republic).

### 4.8. Atomic Force Microscopy (AFM) Imaging and Analysis of Nanomechanical Properties of Bacterial Surface

The bacteria were prepared essentially as described in our previous papers [65] with some modifications. Briefly, the log-phase bacteria suspended in sterile 20 mM phosphate buffer, pH 6.8 (30 µL, OD_600_ = 0.2) were incubated for 45, 60, and 90 min without (control) and in the presence of 10 μM P1 and P2 peptides at 37 °C (*E. coli*) or 5 μM P1 and P2 peptides at 30 °C (*M. luteus*). After centrifugation (8000× *g*, 10 min, 4 °C), the cells were gently washed with 20 mM phosphate buffer, pH 6.8, and then with pyrogen-free water. Finally, the bacteria suspended in 5 μL of pyrogen-free water were applied on mica disks and dried at 28 °C. A Nanoscope V AFM (Veeco, Plainview, NY, USA) was used for bacteria imaging. The measurements were performed in the PeakForce QNM operation mode using a RTESPA-300 silicon probe with spring constant 20–80 N/m (Bruker Nano Inc. Billerica, MA, USA). For each sample, three fields (5 µm × 5 µm, 1 µm × 1 µm, and 400 nm × 400 nm) were imaged. The average root-mean square (RMS) roughness values and adhesion forces were calculated from forty 200 nm × 200 nm fields, measured on each 1 µm × 1 µm image. The Young modulus values were calculated from 500 nm × 500 nm fields. NanoScope Analysis software ver. 1.40 (Veeco, USA) was used for analysis of the nanomechanical properties of bacterial cell surface. WSxM 5.0 software (Nanotec Electronica S.L., Madrid, Spain) was used to generate the three-dimensional (3D) images and section profile diagrams [66]. Three independent experiments were performed for each bacterial strain.

### 4.9. FTIR Spectroscopy Study

For the FTIR analysis of the P1 and P2 peptides interactions with bacterial cells, 3 μL of the *E. coli* JM83 or *M. luteus* suspension (OD_600_ = 0.2) prepared in PBS, pH 6.8, supplemented with 5% D_2_O (*v*/*v*), was transferred onto the attenuated total reflection (ATR) crystal element after 15-min incubation without (control) and with P1 or P2 peptides (final concentration: 10 μM). The samples were deposited on the ATR crystal element by 5-min evaporation and the absorption spectra of the control cells, cells exposed to P1 or P2, P1 and P2 peptides alone, and PBS were collected. The 5-min period was selected and standardized for all the experiments on the basis of preliminary optimization experiments where IR absorption spectra were recorded repeatedly until no spectral changes were observed in the O–H stretching region. Infrared absorption spectra were recorded using a Fourier transform infrared absorption spectrometer Nicolet iS50R (Thermo Scientific, Waltham, MA, USA) equipped with an attenuated total reflection set-up (ATR–FTIR). Continuous purging with argon during the measurements was applied. Typically, 10 scans were collected, Fourier transformed, and averaged for each measurement. All the spectroscopic experiments were performed at 21 °C. The spectral analysis was performed with Grams Al software 9.0 (ThermoGalactic, Salem, NH, USA).

### 4.10. Statistical Analysis

The statistical analysis was performed using Student’s *t* test. The data were presented as the means ± standard deviation (±S.D.). The differences were considered statistically significant at * *p* < 0.05, ** *p* ≤ 0.01, and *** *p* ≤ 0.001.

## 5. Conclusions

Proline-rich AMPs constitute a family diverse in terms of spectrum of antimicrobial activity and modes of action. Non-membranolytic PrAMPs with activity against Gram-negative bacteria can interact with inner membrane transporters and, once inside the bacterial cells, with intracellular targets, such as the 70S ribosome and/or DnaK proteins, causing inhibition of protein biosynthesis and/or making impossible the proper folding of already synthesized polypeptides. Among the PrAMPs, there are also peptides that exhibit dual mode of action, capable of both penetrating bacterial membranes and interacting with intracellular targets. Membrane-lytic PrAMPs, acting at the level of microbial membranes, have also been characterized.

*G. mellonella* PrAMPs, P1 and P2, demonstrated activity against Gram-negative and Gram-positive bacteria, while P1 was also active against yeast and yeast-like fungi. Both peptides bound to *E. coli* and *M. luteus* cells and disrupted the integrity of bacterial membranes, as evidenced by increased membrane permeability. Analysis of absorption spectra (FTIR spectroscopy) revealed shifts in the protein-to-lipid ratio and conformational changes of proteins in bacterial cells treated with both peptides. The nature of these alterations depended on both the bacteria (*E. coli* versus *M. luteus*) and the peptide properties (P1 versus P2). Both peptides caused evident alterations in the surface topography and nanomechanical properties of *E. coli* and *M. luteus* cells (AFM imaging), which were accompanied by morphology changes and damage to bacterial cells (SEM imaging). The results indicated that the antibacterial action of both *G. mellonella* PrAMPs was related to the disruption of bacterial membrane integrity. These peptides are further examples of natural PrAMPs with microbial membrane permeabilizing properties.

Given the enormous diversity of AMPs, including PrAMPs, occurring in nature, there is still a need for research to understand the mechanisms of action of natural PrAMPs and their derivatives. Such studies may shed new light and provide valuable data enabling better use of natural AMPs as templates in rational drug design, as recently presented by Di Stasi et al. [67], who developed chimeric PrAMPs with enhanced activity against ESKAPEE pathogens, containing fragments of the C-terminal regions of natural PrAMPs, Tur1A, and Lip1 from cetaceans [67].

## Data Availability

The original contributions presented in this study are included in the article/Supplementary Material. Further inquiries can be directed to the corresponding author.

## Supplementary Materials

The following supporting information can be downloaded at: https://www.mdpi.com/article/10.3390/ijms26178438/s1.
