# Supplementary material for: Interactions of Galleria mellonella Proline-Rich Antimicrobial Peptides with Gram-Negative and Gram-Positive Bacteria
Source: Int J Mol Sci. 2025 Aug 29;26(17):8438. doi: 10.3390/ijms26178438 (PMC12428502; doi:10.3390/ijms26178438)
Supplement: Supplementary file 1 [file ijms-26-08438-s001.zip › ijms-3817384-supplement-v1.pdf]

Table S1. Nanomechanical properties of *M. luteus* cell surface after incubation without (C) and with P1 and P2 peptides for 60 minutes.

RMS roughness [nm]			Adhesion forces [nV]			Young Modulus [MPa]		
C	P1	P2	C	P1	P2	C	P1	P2
24.12 (±5.48)	18.43 (±4.42)$^{p=0.003}$	9.589 (±2.62) $^{p=0.002}$	3.59 (±1.98)	1.846 (±0.985)$^{p=0.002}$	3.758 (±2.13)$^{p=0.004}$	3196.33 (±83.74)	1879.12 (±228.35)$^{p=0.012}$	1858.33 (±176.37)$^{p=0.002}$

Table S2. Nanomechanical properties of *E. coli* cell surface after incubation without (C) and with P1 and P2 peptides for 45, 60, and 90 minutes.

Time (min)	RMS roughness [nm]			Adhesion forces [nV]			Young Modulus [MPa]		
	C	P1	P2	C	P1	P2	C	P1	P2
45	1.73 (±0.49)	1.54 (±0.33)$^{p=0.8}$	2.5 (±0.53) $^{p=0.0003}$	0.555 (±0.54)	0.454 (±0.167)$^{p=0.256}$	0.392 (±1.05)$^{p=0.002}$	2262.25 (±258.70)	2065.17 (±80.11)$^{p=0.07}$	1143.34 (±176.2)$^{p=0.0007}$
60	1.2 (±0.22)	1.01 (±0.43)$^{p=0.06}$	1.91 (±0.75) $^{p=0.0008}$	0.145 (±0.14)	0.21 (±0.08)$^{p=0.0001}$	0.139 (±0.12)$^{p=0.76}$	2419.2 (±268.2)	673.78 (±522.3)$^{p=2.74685E-06}$	3585.01 (±1186.9)$^{p=0.343}$
90	2.362 (±0.76)	1.978 (±0.79)$^{p=0.02}$	2.598 (±0.95)$^{p=0.2}$	1.47 (±0.52)	0.728 (±0.198)$^{p=6.08002E-11}$	1.804 (±0.37)$^{p=0.47}$	2387.83 (±234.04)	3159.07 (±1049.62)$^{p=0.02}$	2016.67 (±475.86)$^{p=0,004}$

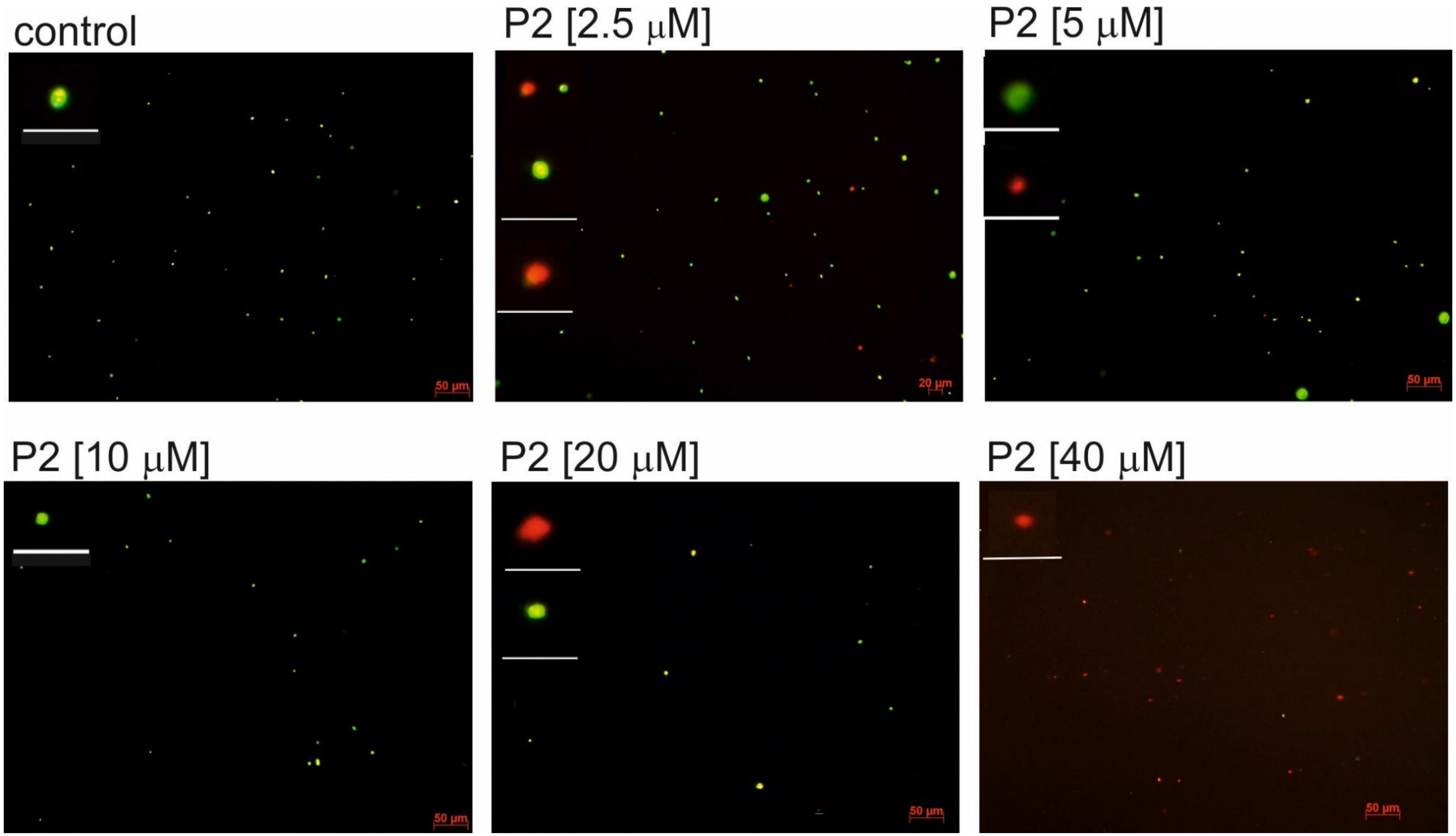

Figure S1. LIVE/DEAD staining of *M. luteus* cells treated with the P2 peptide (LSCM imaging). The live and dead cells are in green and red, respectively.

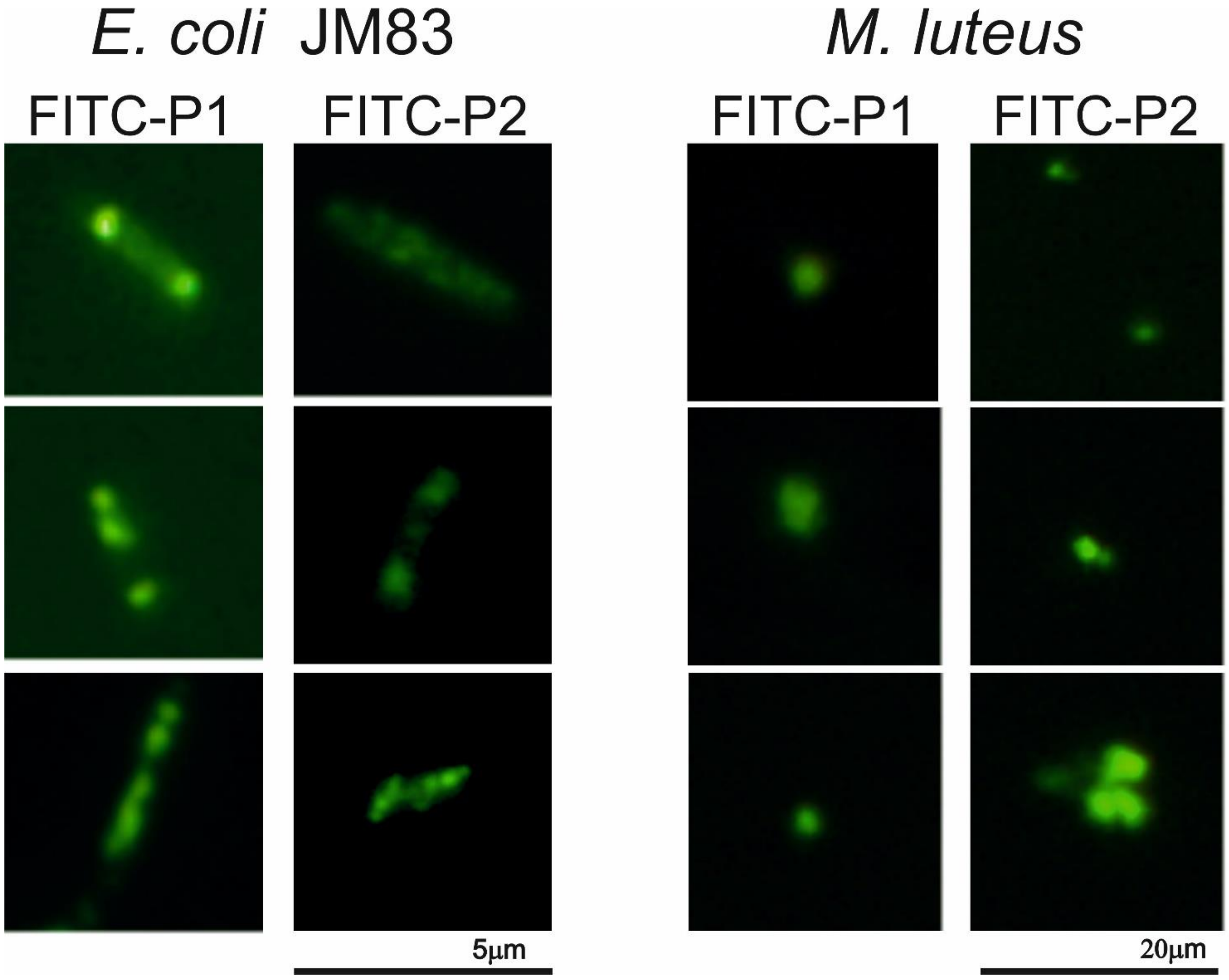

Figure S2. Laser scanning confocal microscopy imaging of FITC-P1 and FITC-P2 binding to *E. coli* and *M. luteus* cells. The bacteria were incubated in the presence of FITC-labeled peptides for 15 minutes and then imaged by LSCM as described in the Section 4.
